# Pediatric emergency department-based asthma education tools and parent/child asthma knowledge

**DOI:** 10.1186/s13223-024-00884-w

**Published:** 2024-03-25

**Authors:** Kina Goodman, Rosa I. Arriaga, Rawan Korman, Farzina Zafar, Cal Stephens, Polly Kumari, Karthika Jayaprakash, Anne M. Fitzpatrick, Nicholas Cooper, Claudia R. Morris

**Affiliations:** 1 Department of Pediatrics, Emory University School of Medicine, Atlanta, GA USA; 2 Pediatric Emergency Medicine Associates, Atlanta, GA USA; 3 Children’s Healthcare of Atlanta, Atlanta, GA USA; 4 Interactive Computing, Georgia Institute of Technology, Atlanta, GA USA; 5 The Wilbur Fisk Glenn Jr. Distinguished Faculty Chair for Clinical & Translational Research, Department of Pediatrics, Division of Pediatric Emergency Medicine, Emory University School of Medicine, Atlanta, GA USA

**Keywords:** Asthma, Asthma education, Health literacy, Pediatric emergency department, Video-game learning

## Abstract

Asthma exacerbations are a leading cause of pediatric hospitalizations despite multiple efforts to educate patients and families on disease course and medication management. Asthma education in the pediatric emergency department (ED) is challenging, and although the use of written action plans has been associated with reduction in hospitalizations and ED visits, written tools may not be useful for individuals with low health literacy. Moreover, asthmatic children should participate in their asthma education. In this prospective randomized study of 53 families presenting to a pediatric ED with a child experiencing an asthma exacerbation, education on asthma was presented via an interactive mobile-based video-game versus a standard-of-care asthma education video (SAV). Median age was 10 years; 64% were males. Many patients had moderate-to-severe asthma, with 57% experiencing ≥ 2 asthma-related ED visits in the last year, 58% requiring hospitalization and 32% reporting a critical care admission. In this cohort, the mobile-based video-game was found to be a feasible, acceptable educational tool; 86% of parents and 96% of children liked the game, while 96% of parents and 76% of children preferred playing the game over watching a SAV. Despite a history of persistent asthma, only 34% of children used an inhaled corticosteroid while 70% required rescue inhaler use in the prior week. Basic asthma knowledge was sub-optimal with only 60% of parents and 43% of children correctly recognizing symptoms that should prompt immediate medical care. This reflects a major gap in asthma knowledge that coexists with parental misconceptions regarding optimal asthma management.

## To the editor

Asthma is the leading cause of pediatric hospitalizations despite multiple efforts to educate patients and their families on disease course and medication management [1]. Asthma education may be challenging in the emergency department (ED) setting, particularly for individuals with low health literacy [2].

To encourage a more engaged and fun learning experience, educational video-games can be useful tools for health education [3, 4], but limited studies have been done in an acute ED setting. Several studies showed increased asthma knowledge, self-management skills, lower asthma symptoms scores, and fewer hospitalizations after utilizing computer-based asthma video-games [5–7]. Video-game learning can help patients and their families better visualize the pathophysiology of asthma, recognize the objectives of its treatments and identify asthma exacerbations in an interactive format that does not require traditional didactic lectures and require minimal reading levels. The objectives of our study are to determine the feasibility and patient/guardian satisfaction of utilizing an interactive mobile-based educational video-game on asthma compared to a standard-of-care asthma educational video (SAV) in a pediatric ED setting in children presenting with acute asthma, and to assess parent/child asthma knowledge after receiving the asthma education intervention. Feasibility is defined by willingness to consent and participate in the study, reflected by the percent of families approached who are ultimately randomized.

A convenience sample of 53 English-speaking families with children previously diagnosed with asthma aged 6–18 years who presented to an urban Pediatric ED (PED) with an acute asthma exacerbation requiring bronchodilator therapy and an oral corticosteroid, and an institutionally-modified clinical respiratory score [8] ≤ 8 were approached for enrollment. Patient families were randomized to play an educational video-game or watch the SAV. The game utilizes augmented reality to enable asthmatic children to be more engaged and interactive in their asthma education. The video-game was uniquely developed to include all critical elements taught in the SAV to represent a comparable model. Following the video-game or SAV, both child and parent asthma knowledge was evaluated using a shortened version of the Newcastle Asthma Knowledge Questionnaire [9] consisting of 11 items assessing knowledge about asthma symptoms, triggers and treatment with a total score range between 0 and 15; higher score indicates greater knowledge. Patient families randomized to the video-game intervention were additionally asked to complete four five-point Likert scale questions that elicited child/parent opinions about the game. In a subset of 30 patients, the Newest Vital Sign (NVS), a nutrition label accompanied by six questions, was utilized to assess health literacy [10]. A total of < 4 correct responses indicate a higher possibility of limited literacy of the participant [10]. For all patients, demographic characteristics collected included patient’s age, gender, grade level and health insurance type. Parent demographics included gender, relation to the child, and the highest level of education attained (Table 1). Parents were questioned about the child’s last use of rescue inhalers, inhaled/oral corticosteroids, ED visits, and hospitalizations. The Emory Institutional Review Board approved the study.

**Table 1:** Demographics, Education, and Asthma clinical history

Variable	Overall *N* = 53	Video Game *N* = 26	Educational Video *N* = 27
**Age- All;** median (25th -75th ) <13 years >=13 years	10 (8, 12)	9.5 (7–12)	10 (8–13)
	40 (75%)	20 (77%)	20 (74%)
	13 (25%)	6 (23%)	7 (26%)
**Sex –** Male; n (%)	34 (64%)	17 (65%)	17 (63%)
**Subject Grade;** n (%) Elementary High school			
	44 (83%)	21 (81%)	23 (85%)
	9 (17%)	5 (19%)	4 (15%)
**Insurance –** Public; n (%)	42 (79%)	23 (88%)	19 (70%)
**CRS (0–12);** median (25th -75th )			
On Admission	3 (2, 5)	3 (2, 4)	3 (2, 5)
On Discharge	2 (1, 2)	1 (1, 2)	2 (1, 3)
**Disposition –** Admit; n (%)	24 (45%)	12 (46%)	12 (44%)
**Parent/Guardian Sex –** Female; n (%)	48 (91%)	25 (96%)	23 (85%)
**Relation to Patient;** n (%)			
Mother	44 (83%)	23 (88%)	21 (78%)
Father	7 (13%)	2 (8%)	5 (18%)
Grandparent	1 (2%)	1 (4%)	0 (0%)
Guardian	1 (2%)	0 (0%)	1 (4%)
**Education of Parent/Guardian**; n (%)			
College Graduate	16 (30%)	7 (27%)	9 (33%)
Graduate Degree	8 (15%)	3 (12%)	5 (19%)
High school diploma	26 (49%)	15 (58%)	11 (41%)
Less than high school	3 (6%)	1 (4%)	2 (7%)
**Asthma Medication use in last 7 days;** n (%)			
Rescue Inhaler	37 (70%)	18 (69%)	19 (70%)
Inhaled Corticosteroid	18 (34%)	7 (27%)	11 (41%)
Oral corticosteroid	7 (13%)	4 (15%)	3 (11%)
**Oral corticosteroid use for asthma (ever);** n (%)	37 (70%)	16 (62%)	21 (78%)
**Last time in ED;** n (%)			
> 2 years ago	9 (17%)	4 (15%)	5(19%)
> 1 year ago	14 (27%)	8 (31%)	6 (22%)
Within 6–12 months	15 (28%)	7 (27%)	8 (29%)
Within 2–6 months	6 (11%)	3 (12%)	3 (11%)
Within 2 months	9 (17%)	4 (15%)	5 (19%)
**Child ED Visits;** n (%)			
No prior visits	0 (0%)	0 (0%)	0 (0%)
1 time	23 (43%)	13 (50%)	10 (37%)
2 times	11 (21%)	6 (23%)	5 (19%)
3 times	11 (21%)	5 (19%)	6 (22%)
>3 times	8 (15%)	2 (8%)	6 (22%)
**Previous Hospitalization – yes;** n (%)	31 (58%)	15(58%)	16 (59%)
# of Admits:			
0 times	0 (0%)	0 (0%)	0 (0%)
1 time	12 (38%)	5 (33%)	7 (44%)
2 times	7 (23%)	4 (27%)	3 (19%)
3 times	3 (10%)	3 (20%)	0 (0%)
> 3 times	9 (29%)	3 (20%)	6 (37%)
**PICU admit;** n (%)	17 (32%)	6 (23%)	11 (41%)

CRS = Clinical Respiratory Score

Medians and 25th-75th percentiles were reported for continuous data and counts, whereas percentages were reported for categorical data. Wilcoxon rank-sum tests and chi-squared tests were used to compare video-game versus SAV groups. The generalized linear model was used to compare Newcastle scores, adjusting for age and NVS. Analysis was completed using SAS 9.4 (Cary, NC).

All 53 patient families approached (*n* = 26 video-game, *n* = 27 SAV) consented and completed the trial, yielding a 100% consent-for-participation rate, demonstrating feasibility. The video-game was well received; 86% of parents and 96% of children liked the game, and 96% of parents and 76% of children preferred it over watching the asthma education video (Table 2). Additionally, 91% of parents and 73% of children reported that the game helped them in understanding how to care for their child’s or their asthma, respectively. Median scores [25th -75th ] on the Newcastle Asthma Knowledge Questionnaire for all enrolled parents (*n* = 53) versus. children (*n* = 53) were 12 [10,14] versus. 10 [8,12]; *p* < 0.001, respectively. There was no difference in the scores between the video-game and SAV groups, suggesting that the video-game provided similar asthma education knowledge.

In our study group, 83% of parent participants were mothers, 13% were fathers, and 4% were grandparent/guardian. This highlights a significant gap regarding limited paternal education in asthma care in an acute setting that is essential in improving asthma knowledge across the family.

Of the 30 parents that completed the health literacy tool (NVS), 47% had < 4 questions correct, indicating limited literacy. Since this may impact comprehension of standard asthma education, this further highlights the importance of utilizing education tools that are more accommodating and clearer for families with limited literacy. When asked about asthma care the week before, 70% of children required rescue inhaler use, but only 34% had used an inhaled corticosteroid. This is alarming given that 57% of patients had two or more ED visits in the last year for asthma; 58% required hospitalization and 32% reported a previous pediatric intensive care unit (PICU) admission. While the etiology of this concerning phenomenon cannot be determined by this study, it is likely a combination of poor adherence to asthma medications, inhaled steroid ‘addiction’ misconceptions, and low adherence to the National Heart, Lung and Blood Institute (NHLBI) asthma guidelines by primary care providers who prescribe asthma rescue medications without a controller medication.

Table 2 summarizes parent/patient asthma knowledge, Newcastle Score, and video-game satisfaction. All parents recognized that second-hand smoke worsens asthma. However, when it came to acute recognition of the need for medical intervention, only 60% of parents and 43% of children recognized that a rescue inhaler used every 2–3 h should prompt immediate medical care. Even fewer families recognized the use of a spacer to aid in inhaled medication delivery. This highlights gaps in understanding basic asthma knowledge and translating it to acute asthma management. Furthermore, 75% of parents and 54% of children thought that inhaled corticosteroids were addictive (Table 2). This may contribute to the low percentage (34%) of patients in our cohort on an inhaled corticosteroid. This is even more concerning given that nearly a third of our participants required a prior PICU stay suggestive of severe asthma. More education needs to be focused on daily asthma control through inhaled corticosteroid use. This includes dispelling myths associated with inhaled corticosteroids and the benefits of their use.

This study had several limitations. First, there are biases inherent to surveys such as social desirability bias which may skew the true acceptability of our intervention. Additionally, our study was only offered in English and is a single-center study with a relatively small sample size, limiting generalizability.

**Table 2:** Parent/Patient Asthma Knowledge after watching the standard-of-care asthma educational video or playing the asthma education video-game, Newcastle Score and Video Game Satisfaction

Variable	Correct Answer	Parent (*N* = 53)	Child (*N* = 53)	*P-Value*
*Select Newcastle Questions (out of 11 questions):				
Asthma can be cured	False	74%	56%	0.06
Asthmatics can become addicted to their medications	False	75%	54%	**0.03**
Secondhand smoke can make asthma worse	True	100%	90%	**0.024**
With the right treatment, children with asthma can live a normal life	True	98%	78%	**0.001**
Timmy wakes up at night with cough 2 days a week, is his asthma in control?	No	87%	78%	0.24
Is it ok if you need to use your inhaler every 2–3 h while at home?	No	60%	43%	0.08
**Overall Newcastle Asthma Knowledge Score**; Median (25^th^ -75^th^)	Score 0–15	12 (10,14)	10 (8,12)	**< 0.001**
		*N* = 22	*N* = 26	
**Video Game Satisfaction Likert Score Overall Score.** Median (25th -75th ) **Survey Items**:	(1–5 Scale) 1: Strongly Disagree 5: Strongly Agree	4 (4,5)	4 (4,5)	0.38
1) I liked the game.		4 (4–5)	4 (4–5)	
2) I found the game helpful in understanding how to care for my asthma.		4 (4–5)	4 (3–5)	
3) I would rather play the game than watch an asthma education video.		4 (4–5)	5 (4–5)	
4) I would recommend this game to others.		4 (4–5)	4 (4–5)	

Chi-squared tests and Wilcoxon rank-sum tests comparing parent and child responses (treated as independent groups)

*Data reflect % of Parent and Child providing correct true/false or yes/no answer

In conclusion, our novel intervention using an interactive video-game for ED-based asthma education, that could also be easily used at home and different settings, was feasible and accepted by both children and their families, with high parent/child satisfaction. Despite relatively high parent education level and overall scores on the knowledge tool, false beliefs, and lack of understanding of proper asthma management were identified. There was an overall poor recognition of progressing asthma symptoms that need an urgent ED evaluation, and the majority of parents/children believed that asthma medications are addictive. These misconceptions could greatly impact asthma management. Identifying gaps in parent/child asthma knowledge is essential to improving asthma education. Further research is warranted.

## Data Availability

Data analyzed for this study can be made available upon sending a reasonable request to the corresponding author.

## Abbreviations

NVS: Newest Vital Sign
PED: Pediatric Emergency Department
SAV: Standard Asthma Video
